# Commentary: The Effect of Systemic Nitroglycerin Administration on the Kynurenine Pathway in the Rat

**DOI:** 10.3389/fneur.2017.00518

**Published:** 2017-09-29

**Authors:** Marcelo Moraes Valença

**Affiliations:** ^1^Neurology and Neurosurgery Unit, Federal University of Pernambuco, Recife, Brazil

**Keywords:** kynurenine pathway, headache, physiopathology, NMDA receptor, caudal trigeminal nucleus, migraine, cluster headache

A significant role is exerted by glutamate in the physiopathogenesis of migraine. Biological products originated in kynurenine pathway (KP) of tryptophan metabolism may interact with the glutamatergic transmission. Interaction between the KP and the nitrergic system was demonstrated by Nagy-Grócz and coworkers (1). They made a study evaluating the expression of *N*-formyl-l-kynurenine by tryptophan 2,3-dioxygenase (TDO2), indoleamine 2,3-dioxygenase (IDO1), anthranilic acid (ANA) by l-kynurenine hydrolase (KYNU), and 3-hydroxykynurenine (3-HK) by l-kynurenine 3-monooxygenase (KMO) in the brain after intraperitoneal administration of nitroglycerin. As a matter of fact, only enzyme levels were reported, not the products of the enzymes. Nitroglycerin significantly diminished the expression of TDO2, IDO1, KYNU, and KMO in the trigeminal nucleus caudalis (TNC) (1).

This indicates that the KP may be one of the several mechanisms through which a headache attack could be triggered or attenuated. Different compounds originating in the KP may have distinct physiological functions. Activation of the KP may produce compounds that can inhibit NMDA receptors, which may be involved in migraine chronification or triggering headache crises. On the contrary, the inhibition of this pathway would allow a more effective interaction of glutamate with NMDA receptors. With this in mind, it is important to note that one of the key metabolites in the KP is kynurenic acid, an endogenous glutamate receptor antagonist. Key products of the KP may act at cross-purposes—an agonist and an antagonist at the NMDA receptor. A very intriguing possibility, however, that I would like to mention is a very selective control of each of the enzymatic steps in such a way as to promote a differentiated synthesis of KP metabolites, as in the example of the catecholaminergic neurons. This would lead to the predominance of some KP metabolite(s) in relation to others under different physiological or pharmacological conditions. The absence of some enzymes in different cells would, nonetheless, produce NMDA agonist neuroactive substances or *vice versa*.

Such KP metabolites, originating from a cascade of enzymatic steps, can bind to different receptors. For example, kynurenine binds through an aryl hydrocarbon receptor. Quinolinic acid is an agonist at NMDA receptors. Kynurenic acid, in addition to blocking NMDA receptors and α7-homomeric nicotinic cholinoceptors, also acts as an agonist at the orphan G-protein-coupled receptor 35 (GPR35). And cinnabarinic acid activates metabotropic glutamate receptors (2). One possible CNS site in which this may occur is the TNC, which receives peripheral afferents from the trigeminal system, an area where upregulation of Fos occurs after nitroglycerin treatment (3). An increase in nNOS and CGRP in the dura mater and CGRP in the TNC was also observed after nitroglycerin administration, which was inhibited by olcegepant, an antagonist of CGRP receptors (3). This suggests an interaction between nitroglycerin and CGRP system in TNC. Also, evidence indicates that there are bidirectional interactions between the KP and NO production in the brain.

Nitroglycerine, when administered to migraineurs, is able to trigger a headache with characteristics similar to those of migraine attacks (4), strongly indicating that the production of NO is involved in a migraine crisis. Previously, we measured the serum NO levels in 357 women during a migraine attack (5, 6). The levels were significantly higher during the migraine attack than at times when they were without pain (5, 6). Interestingly, preventive treatment with either propranolol or amitriptyline induced a significant decline in the serum levels of NO after 2, 4, and 6 months (5, 6). In addition, simvastatin, a drug known to inhibit inductive NO synthase and stimulate endothelial NO synthase, also decreased the NO concentration in the serum, an effect associated with a significant decline in the frequency of migraine attacks in dyslipidemic women (5, 6).

Thus, NMDA receptors can be activated or inhibited by biologically active molecules generated by tryptophan metabolism through the KP and this seems to play a role in several primary headache disorders, such as migraine and cluster headache (CH) (7–9). Martelletti and coworkers (8) were able to measure kynurenine, kynurenic acid, ANA, 3-hydroxy-anthranilic acid (3-HANA), 3-HK, xanthurenic acid (XA), quinolinic acid, tryptophan, and 5-hydroxyindoleacetic acid (5-HIAA) in the serum of CH patients. Significant decreases were observed in kynurenine, kynurenic acid, 3-HK, 3-HANA, XA, 5-HIAA, and quinolinic acid compared to a control group. An increase was observed in the concentrations of tryptophan and ANA. Interestingly, no differences were identified in the levels of any of the abovementioned metabolites when comparing episodic and chronic CH patients, with the exception of kynurenine levels, which were higher in those with chronic CH (8). This indicates that the KP products may be produced and released in a differentiated form, suggesting selective activation of KP enzymes.

Recent evidence has demonstrated that l-kynurenine or one of its derivatives may regulate the systemic circulation, among other things by dilating arterial blood vessels and increasing blood flow to the brain (10). Migraine is a neurologic disorder with major involvement of blood vessels, probably exerting a role in the genesis of pain felt by the patient. Thus, activation or inhibition of KP may be part of the vascular phenomena (e.g., cortical spreading oligemia, blood pressure changes, and facial pallor) clearly observed during a migraine attack.

In migraine physiopathology, a large number of putative biologically active molecules participate at different anatomical regulatory sites, e.g., dura mater, trigeminal ganglia, CNS, and head vessels. Enzymes and specific receptors are activated or inhibited during the process of this important alarm system, signaling a potential internal or environmental danger. The therapeutic arsenal currently used was developed with the aim of targeting some of these control levels by stimulating or inhibiting specific receptors or enzymes. A better understanding how the KP and its enzymes work in different headache subtypes will probably lead to the development of new and more effective drugs for use in either acute or preventive treatment.

In conclusion, the authors (1) used a rodent model to further study the mechanisms through which primary headaches are triggered. Interestingly, all four investigated enzymes underwent downregulation when the animal was treated with NGT, a drug known to cause headache in humans. Further studies are required to demonstrate whether each of these enzymes could be regulated in an independent or specific manner.

## Author Contributions

The author confirms being the sole contributor of this work and approved it for publication.

## Conflict of Interest Statement

The author declares that the research was conducted in the absence of any commercial or financial relationships that could be construed as a potential conflict of interest.

## References

[1] Nagy-Grócz G Laborc KF Veres G Bajtai A Bohár Z Zádori D The effect of systemic nitroglycerin administration on the kynurenine pathway in the rat. Front Neurol (2017) 8:278 10.3389/fneur.2017.00278 28659861 PMC5469907

[2] Stone TW Stoy N Darlington LG. An expanding range of targets for kynurenine metabolites of tryptophan. Trends Pharmacol Sci (2013) 34(2):136–43. 10.1016/j.tips.2012.09.006 23123095

[3] Ramachandran R Bhatt DK Ploug KB Hay-Schmidt A Jansen-Olesen I Gupta S Nitric oxide synthase, calcitonin gene-related peptide and NK-1 receptor mechanisms are involved in GTN-induced neuronal activation. Cephalalgia (2014) 34(2):136–47. 10.1177/0333102413502735 24000375

[4] Ashina M Hansen JM Olesen J. Pearls and pitfalls in human pharmacological models of migraine: 30 years’ experience. Cephalalgia (2013) 33(8):540–53. 10.1177/0333102412475234 23671251

[5] Medeiros FL Medeiros PL Reis WL Antunes-Rodrigues J Valença MM Migraine prevention with low doses of propranolol and amitriptylin: correlation with nitric oxide production. 14th Congress of the International Headache Society, 2009, Philadelphia (Vol. 29), Oslo: Cephalalgia, Blackwell Publishing (2009). p. 36–36.

[6] Medeiros FL Medeiros PL Valença MM Dodick D Simvastatin for migraine prevention. Headache (2007) 47(6):855–6. 10.1111/j.1526-4610.2007.00824.x 17578535

[7] Tajti J Szok D Nagy-Grócz G Tuka B Petrovics-Balog A Toldi J Kynurenines and PACAP in migraine: medicinal chemistry and pathogenetic aspects. Curr Med Chem (2017) 24:1332–49. 10.2174/0929867324666170227115019 28245765

[8] Curto M Lionetto L Negro A Capi M Perugino F Fazio F Altered serum levels of kynurenine metabolites in patients affected by cluster headache. J Headache Pain (2015) 17:27. 10.1186/s10194-016-0620-2 27000870 PMC4801826

[9] Curto M Lionetto L Negro A Capi M Fazio F Giamberardino MA Altered kynurenine pathway metabolites in serum of chronic migraine patients. J Headache Pain (2015) 17:47. 10.1186/s10194-016-0638-5 27130315 PMC4851673

[10] Varga DP Menyhárt Á Puskás T Bari F Farkas E Kis Z Systemic administration of l-kynurenine sulfate induces cerebral hypoperfusion transients in adult C57Bl/6 mice. Microvasc Res (2017) 114:19–25. 10.1016/j.mvr.2017.05.006 28546077

